# Loneliness is adversely associated with physical and mental health and lifestyle factors: Results from a Swiss national survey

**DOI:** 10.1371/journal.pone.0181442

**Published:** 2017-07-17

**Authors:** Aline Richard, Sabine Rohrmann, Caroline L. Vandeleur, Margareta Schmid, Jürgen Barth, Monika Eichholzer

**Affiliations:** 1 Epidemiology, Biostatistics and Prevention Institute, Division of Chronic Disease Epidemiology, University of Zurich, Hirschengraben 84, Zurich, Switzerland; 2 Centre for Research in Psychiatric Epidemiology and Psychopathology, Department of Psychiatry, University Hospital of Lausanne, Site de Cery, Prilly, Switzerland; 3 Institute for Complementary and Integrative Medicine, University Hospital and University of Zurich, Sonneggstr. 6, Zurich, Switzerland; Cardiff University, UNITED KINGDOM

## Abstract

**Introduction:**

Loneliness is a common, emotionally distressing experience and is associated with adverse physical and mental health and an unhealthy lifestyle. Nevertheless, little is known about the prevalence of loneliness in different age groups in Switzerland. Furthermore, the existing evidence about age and gender as potential effect modifiers of the associations between loneliness, physical and mental health and lifestyle characteristics warrants further investigation. Thus, the aim of the study was to examine the prevalence of loneliness among adults in Switzerland and to assess the associations of loneliness with several physical and mental health and behavioral factors, as well as to assess the modifying effect of sex and age.

**Methods:**

Data from 20,007 participants of the cross-sectional population-based Swiss Health Survey 2012 (SHS) were analyzed. Logistic regression analyses were used to assess associations of loneliness with physical and mental health or lifestyle characteristics (e.g. diabetes, depression, physical activity). Wald tests were used to test for interactions.

**Results:**

Loneliness was distributed in a slight U-shaped form from 15 to 75+ year olds, with 64.1% of participants who had never felt lonely. Lonely individuals were more often affected by physical and mental health problems, such as self-reported chronic diseases (Odds ratio [OR] 1.41, 95% confidence interval [CI] 1.30–1.54), high cholesterol levels (OR 1.31, 95% CI 1.18–1.45), diabetes (OR 1.40, 95% CI 1.16–1.67), moderate and high psychological distress (OR 3.74, 95% CI 3.37–4.16), depression (OR 2.78, 95% CI 2.22–3.48) and impaired self-perceived health (OR 1.94, 95% CI 1.74–2.16). Loneliness was significantly associated with most lifestyle factors (e.g. smoking; OR 1.13, 95% 1.05–1.23). Age, but not sex, moderated loneliness’ association with several variables.

**Conclusion:**

Loneliness is associated with poorer physical and mental health and unhealthy lifestyle, modified by age, but not by sex. Our findings illustrate the importance of considering loneliness for physical and mental health and lifestyle factors, not only in older and younger, but also in middle-aged adults. Longitudinal studies are needed in Switzerland to elucidate the causal relationships of these associations.

## Introduction

Throughout decades loneliness has commonly been described by philosophers, psychologists and in the literature as ubiquitous and even as an essence of human being [1]. Loneliness is defined as an emotional distress experience that goes along with the perception of unsatisfying social relationships. Perceived unsatisfying relationships, thus, are independent of the quantity of social interactions, but are caused by the felt sense of social isolation and unsatisfied need for affection in current relationships [2, 3]. Nevertheless, a low quantity and diminished meaning of social contacts has also been related to loneliness.

Sociodemographic characteristics are partly associated with loneliness. In general women seem to report loneliness more frequently than men [4–6], although the gender differences may disappear when controlling for other factors such as widowhood, depression, mobility problems, age, education, and social contacts [7]. Concerning age, some authors have described a U-shaped prevalence with more lonely subjects in younger and older adults than in middle-aged subjects [3, 8, 9]. However, other authors suggested that loneliness is more common in older age [10–13]. Luhmann and Hawkley [14] observed an even more complex association in their nationally representative German sample with an age range from 18 to 103 years. Loneliness levels (measured by a short version of the UCLA Loneliness Scale [15]) showed two peaks around ages 30 and 60 and two dips around ages 40 and 75. There was a general downward trend in loneliness in young and middle-aged adults. The lowest level was found around age 75 after which the levels rose continuously into old age. Overall, most studies focused on older age [7, 16–22], children [23], adolescents and young adults [14, 24], and less is known about the prevalence of loneliness in middle-aged adults [14, 25, 26].

Other sociodemographic factors have been suggested to be risk factors for loneliness, such as being single and/or living alone [8, 25, 27], low educational or low-income levels [16, 28, 29], immigration status, especially immigration in women [30], as well as low social support [31]. In contrast, the fact of living in an urban or rural area as associated with loneliness has yielded contradictory results [16, 28, 32].

Loneliness can be a transient state, but more importantly, it can also be a long-lasting perception [9] with negative health and behavioral outcomes [33–35]. Loneliness is associated with increased morbidity and mortality risk [19, 36]. There is increasing evidence that lonely individuals feel less healthy [37], have a higher risk for hypertension [38], hypercholesterolemia, the metabolic syndrome [19], and coronary heart disease [39]. Furthermore, loneliness has been related to mental health problems, such as psychosis [40, 41], suicide [42], and depression [3, 43]. Loneliness is associated with a lower capacity for self-regulation [3]. As a consequence, lower self-regulation goes along with a worsened lifestyle: Lonely individuals are less physically active [44], are more often affected by alcohol abuse [45] and are more often obese [46] than non-lonely persons.

In 2011, a study compared loneliness in 25 European countries and a north-east gradient for self-reported loneliness was observed [13]. In Northern Europe, including Switzerland, loneliness was under 6%, in contrast to higher prevalences in Russia and Eastern European (about 10–34% for different age groups). In Switzerland, an updated population-based estimate of loneliness frequencies is nevertheless necessary.

In relation to studies evaluating the associations of loneliness with health factors, many of them are of North American origin, but there are also a number of studies which were carried out in Asia, Europe, and New Zealand [3, 35]. In contrast, in Switzerland, the evidence for the associations of loneliness with health and lifestyle factors is very limited [47]. In contrast to several other countries, a rather wealthy country with a universal health care system. Thus, associations between loneliness and health outcomes may differ from other Western countries with different health care systems such as the US. Furthermore, in regard to implement prevention programs in Switzerland, which aim to address loneliness, it is of importance to provide data also for Switzerland. Against this background, we aimed to determine the prevalence of loneliness in a large representative sample of the Swiss population aged 15 years and older, to evaluate loneliness and its associations with health and lifestyle outcomes, with a special emphasis on sex and age as modifiers of these associations.

## Methods

### Study population and data

Data were obtained from the Swiss Health Survey (SHS) conducted in 2012/2013 by the Swiss Federal Bureau of Statistics (SFSO) (Legal basis: Ordinance of the Conduct of Federal Statistical Surveys of 30 June 1993). This cross-sectional, population-based nationwide survey focuses on health status, several lifestyle and demographic factors, and healthcare use and has been carried out every five years since 1992. It uses a stratified random sampling technique based on registries of inhabitants, and with the cantons of Switzerland as strata. Out of an initial sample of 41,008 individuals a total number of 21,597 individuals aged 15 years or older and living in a private household agreed to participate in the 2012 SHS (i.e. participation rate 54%). They participated in a computer-assisted telephone interview (CATI). In a next step, a written questionnaire was provided (paper or online) upon approval from the participants (n = 18,357). The written questionnaire was an extension of the basic telephone interview, with the aim to gather more detailed information on some topics, such as dental care, clinical depression, etc. The representativeness of the Swiss population was ensured by the multistage probability sampling and the appropriate weighting factors provided by the SFSO. The sampling weights provided by the SFSO were used which allows a comparison with the permanent 2012 Swiss population regarding sex, age, geographic region and nationality (Swiss vs. others).

From the telephone interview, 20,841 individuals had information available on loneliness. After excluding individuals with missing information on health outcomes listed in Table 1, data on 20,405 individuals were available. In a further step, we excluded individuals with missing information on lifestyle factors (n = 398), and our final sample therefore consisted of n = 20,007 individuals. One exception was the outcome variable “depression” assessed by the Patient Health Questionnaire (PHQ-9), which stems from the written questionnaire (n = 18,357). Out of the 20,007 included individuals only 16,114 individuals had available information on depression status. Thus, results concerning depression included less individuals than the other results.

**Table 1:** Characteristics of the sample of the 2012 Swiss Health Survey^1^.

		Total
Total, n		20,007
Age, mean (SE)		47 (0.17)
		*%*
**Socio-demographics**		
Sex	males	49.1
	females	50.9
Age (years)	≥ 15 to < 30	21.1
	≥ 30 to < 40	15.9
	≥ 40 to < 50	20.3
	≥ 50 to < 60	15.8
	≥ 60 to <70	13.2
	≥ 70	13.7
Area of residence	Urban	72.9
	Rural	27.1
Nationality	Swiss	78.9
	Non-Swiss	21.1
Educational level	High	29.7
	Middle	54.4
	Low	16.0
Marital status	Married / registered partnership	51.0
	Single, divorced / dissolved partnership, separated, widowed	49.0
Household size	Living alone	13.2
Social support	High	45.2
	Middle	49.7
	Low	1.2
	Not known / no answer	3.9
**Physical and mental health**		
Chronic disease^2^	No	68.8
	Yes	31.2
Hypertension	No	73.9
	Yes	26.1
High cholesterol	No	82.8
	Yes	17.2
Diabetes	No	95.7
	Yes	4.3
Psychological distress^3^	Low	82.1
	Moderate, high	17.9
Depression^4^	No	95.4
	Yes	4.6
Self-perceived health	Good, very good	84.1
	Fair, poor, very poor	15.9
Visit to a physician within the past year	No	21.7
	Yes	78.3
Body mass index	Underweight (BMI < 18.5 kg/m^2^)	3.5
	Normal weight (BMI ≥ 18.5 to < 25.0 kg/m^2^)	55.8
	Overweight (BMI ≥ 25 to < 30.0 kg/m^2^)	30.6
	Obesity (BMI ≥ 30 kg/m^2^)	10.0
**Lifestyle factors**		
Smoking status	Never smokers	49.9
	Ex-smokers	21.7
	Current smokers	28.4
Chronic alcohol consumption^5^	No	95.2
	Yes	4.8
Binge drinking (6 glasses or more on one occasion)	No (less than once a year)	66.7
	Yes (more than once a year)	33.3
Physical activity	≥ 150 minutes per week	73.0
	< 150 minutes per week	27.0
Diet awareness	Yes	69.0
	No	31.0
Adherence to the recommendation of fruit and vegetable consumption	Yes	11.1
	No	88.9
Loneliness	Never	64.1
	Sometimes	31.7
	Quite often	2.7
	Very often	1.5

^1^ Weighted according to the Swiss general population

^2^ Ongoing disease or health problem lasting for at least 6 months or expected to last for longer than 6 months

^3^ Measured by the 5-item mental health index

^4^ Measured by the PHQ-9 in the written questionnaire

^5^ Ethanol ≥ 20 g/day for women, ≥ 40 g/day for men

The SHS was conducted by the Swiss Federal Bureau of Statistics and does not require formal approval by an ethics committee. This data collection is specifically permitted under Swiss la (SR 431.012.1 and SR 431.112.1).

### Measurements

Loneliness was used as a predictor variable and assessed by one item of the SHS asking the participants: “How often do you feel lonely? “Very often”, “quite often”, “sometimes”, “never”. Answers were dichotomized into never vs. sometimes, quite often and very often.

Outcome variables were divided into two parts, first into physical and mental health, and second into lifestyle factors. Physical and mental health included self-reported non-specified chronic diseases (self-reported ongoing disease or health problem lasting for at least 6 months or expected to last for longer than 6 months vs. none). Further health outcomes were self-reported diagnoses of hypertension, and high cholesterol concentration (yes vs. no), diabetes, and psychological distress (low vs. moderate and high) during the previous 4 weeks (assessed by the 5-item mental health index MHI-5). The MHI-5 has been shown to be a valid tool for detecting psychological distress in the general population and in psychiatric surveys [48–51]. From the written questionnaire the validated PHQ-9 was used to detect depression (yes vs. no). We used a cut-off point of ≥10 vs. less, which has been shown to have a sensitivity of 88% and a specificity of 88% for the diagnosis of major depression [52, 53]. Additional health outcomes were self-perceived health (good and very good vs. fair, poor and very poor), having visited a medical doctor within the last year (yes vs. no), and body mass index (BMI) dichotomized into overweight and obese (BMI ≥25.0 kg/m^2^) vs. less. For adolescents aged 15 to 18 years, tables provided by Cole and colleagues were used to define overweight and obesity [54].

Lifestyle included smoking status (never and former vs. current), chronic alcohol consumption associated with a health risk (women ≥20 g, men ≥40 g ethanol daily vs. less), binge drinking (≥ 6 glasses of alcohol at one occasion within the last year vs. less often), physical activity (≥ 150 minutes per week vs. less) [55], diet awareness ("Do you pay attention to specific aspects of your diet?"; yes vs. no), and keeping to the 5-a-day recommendation of fruit and vegetable consumption according to the recommendations of the Swiss Society for Nutrition [56] (yes vs. no).

Confounders were chosen a priori from the known literature and included sex, age (categorized into 5-year age groups), area of residence (urban vs. rural), nationality (Swiss vs. non-Swiss), educational level (low: compulsory education or less vs. middle: secondary education vs. high: tertiary education), marital status (married/registered partnership vs. single, divorced/dissolved, separated, widowed), household size (living alone vs. not living alone), and level of social support received (high, middle, low; based on the Oslo-3-social support scale [57]).

### Statistical analyses

All statistical analyses were conducted using STATA software version 13.1 (College Station, Texas). Weighted percentages were used to illustrate the prevalence of loneliness, socio-demographics, health and lifestyle outcomes. The prevalence of loneliness was shown in 5-year age groups. For further analyses, three age groups (15–29, 30–59, and 60+ years) were built, based on the prevalence results, which showed peaks of loneliness at the ages of 30 and 60 years.

Logistic regression analyses were used to determine associations of loneliness with physical and mental health and lifestyle factors in three models (model 1 unadjusted, model 2 age- and sex adjusted, and model 3 adjusted for age, sex, area of residence, nationality, educational level, marital status, household size, and social support). Odds ratios (OR) with the corresponding 95% confidence intervals (95% CI) were computed. Sampling weights were used for all analyses. Furthermore, a sub-analysis with loneliness dichotomized into extreme groups (feeling never vs. quite/very often lonely) was performed and associations with physical and mental health and lifestyle factors were analyzed.

As for “marital status” and “living alone” no categorization based on recommendations or validated instruments are known, we performed additional sub-analyses with different categorizations, such as without grouping different marital status. This did not affect the results (data not shown). Therefore, we used our dichotomized variables.

P-values < 0.05 were considered to be statistically significant.

We tested for multiplicative interactions by including a cross-product term for indicators of physical and mental health or lifestyle factors, and sex or age (in 3 groups) in the logistic regression models, along with the main effect terms for each. The statistical significance of the coefficient for each cross-product term was evaluated using the Wald test.

## Results

### Description of the sample and frequency of loneliness

**Fig 1:**
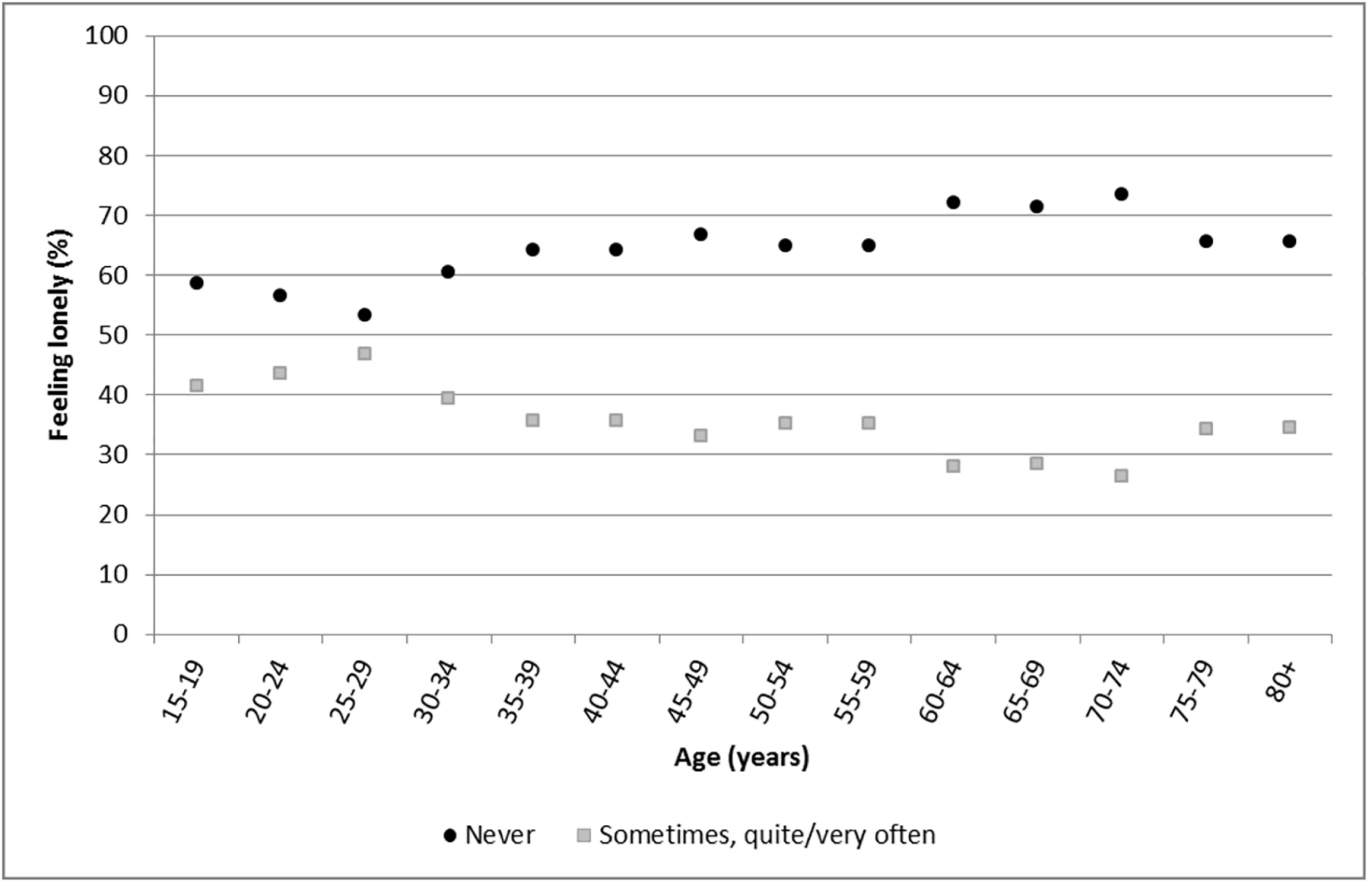
Loneliness in different age groups. Prevalence of perceived frequency of loneliness by 5-year age groups.

Characteristics of the sample as well as the frequencies of loneliness are provided in Table 1. A description of the perceived frequency of loneliness in 5-year age steps from 15 to 80 years or older is shown in Fig 1. In all age groups, between 26% and 47% of individuals felt sometimes, quite or very often lonely. Furthermore, feeling lonely was reported more often by younger individuals than by older ones, with a peak at around 25 to 29 years of age, and a decrease from 70 to 74 years of age followed by a small increase thereafter (Fig 1).

### Loneliness and physical and mental health

Table 2 shows the multivariable adjusted associations between loneliness and physical and mental health or lifestyle factors. Loneliness was statistically significantly associated with self-reported chronic diseases (OR 1.41, 95% CI 1.30–1.54), high cholesterol concentration (OR 1.31, 95% CI 1.18–1.45), diabetes (OR 1.40, 95% CI 1.16–1.67), moderate to high psychological distress (OR 3.74, 95% CI 3.37–4.16), depression (OR 2.78, 95% CI 2.22–3.48), impaired self-perceived health (OR 1.94, 95% CI 1.74–2.16), and with having had contact more often with physicians during the past 12 months (OR 1.29, 95% CI 1.17–1.42), No associations were observed between loneliness and hypertension or loneliness and BMI.

**Table 2:** Associations between loneliness and lifestyle or health-related factors of the 2012 Swiss Health Survey^1^.

		unadjusted		age and sex adjusted		multivariable adjusted	
		*OR*	*95% CI*	*OR*	*95% CI*	*OR*	*95% CI*
**Physical and mental health**							
Chronic disease^2^	No	1		1		1	
	Yes	**1.26**	**[1.17,1.37]**	**1.42**	**[1.31,1.54]**	**1.41**	**[1.30,1.54]**
Hypertension	No	1		1		1	
	Yes	**0.89**	**[0.82,0.97]**	**1.09**	**[1.00,1.20]**	1.07	[0.97,1.17]
High cholesterol	No	1		1		1	
	Yes	1.04	[0.95,1.14]	**1.33**	**[1.20,1.47]**	**1.31**	**[1.18,1.45]**
Diabetes	No	1		1		1	
	Yes	1.06	[0.89,1.25]	**1.40**	**[1.18,1.67]**	**1.40**	**[1.16,1.67]**
Psychological distress^3^	Low	1		1		1	
	Moderate, high	**4.12**	**[3.74,4.55]**	**4.05**	**[3.66,4.47]**	**3.74**	**[3.37,4.16]**
Depression^4^	No	1		1		1	
	Yes	**3.43**	**[2.75,4.28]**	**3.09**	**[2.47,3.87]**	**2.78**	**[2.22,3.48]**
Self-perceived health	Good, very good	1		1		1	
	Fair, poor, very poor	**1.81**	**[1.65,1.99]**	**2.13**	**[1.92,2.36]**	**1.94**	**[1.74,2.16]**
Visit to a physician within the past year	No	1		1		1	
	Yes	**1.34**	**[1.22,1.47]**	**1.26**	**[1.15,1.39]**	**1.29**	**[1.17,1.42]**
Body mass index	BMI < 25.0 kg/m^2^	1		1		1	
	BMI ≥ 25.0 kg/m^2^	**0.76**	**[0.70,0.82]**	0.94	[0.87,1.02]	0.93	[0.86,1.01]
**Lifestyle factors**							
Smoking status	Never and ever smokers	1		1		1	
	Current smokers	1.06	[0.99,1.14]	**1.18**	**[1.09,1.27]**	**1.13**	**[1.05,1.23]**
Chronic alcohol consumption^5^	No	1		1		1	
	Yes	0.99	[0.84,1.16]	1.04	[0.88,1.23]	1.03	[0.87,1.22]
Binge drinking	No			1			
	Yes	1.01	[0.93,1.09]	1.05	[0.96,1.15]	1.09	[0.99,1.19]
Physical activity	≥ 150 minutes per week	1		1		1	
	< 150 minutes per week	**1.30**	**[1.20,1.41]**	**1.31**	**[1.21,1.43]**	**1.20**	**[1.10,1.31]**
Diet awareness	Yes	1		1		1	
	No	0.97	[0.89,1.05]	0.99	[0.91,1.08]	0.97	[0.89,1.06]
Adherence to the recommendation of fruit and vegetable consumption	Yes	1		1		1	
	No	**1.13**	**[1.00,1.27]**	**1.32**	**[1.17,1.49]**	**1.21**	**[1.07,1.37]**

^1^ Weighted according to the Swiss general population and adjusted for age, sex, area of residence, nationality, educational level, marital status, household size and social support

^2^ Ongoing disease or health problem lasting for at least 6 months or expected to last for longer than 6 months

^3^ Measured by the 5-item mental health index

^4^ Measured by the PHQ-9 in the written questionnaire

^5^ Ethanol ≥ 20 g/day for women, ≥ 40 g/day for men

### Loneliness and lifestyle

Compared to participants who never felt lonely, lonely men and women were statistically significantly more frequently current smokers (OR 1.13, 95% CI 1.05–1.23), less physically active (OR 1.20, 95% CI 1.10–1.31), and adhered less often to the recommendations of fruit and vegetable consumption (OR 1.21, 95% CI 1.07–1.37) (Table 2, multivariable adjusted for sociodemographic and social support variables in Table 1). Loneliness was not associated with chronic alcohol consumption, binge drinking, or diet awareness.

We only observed one statistically significant interaction of loneliness with sex, for diet awareness (p-interaction < 0.001, data not shown). Indeed, lonely men mentioned statistically significantly more awareness to diet than men who never felt lonely, but there was no such association between loneliness and diet in women.

Associations were even stronger in the sub-analysis, in which never lonely individuals were compared to quite and very often lonely individuals, with one exception, i.e. the association of loneliness with smoking was no longer statistically significant (data not shown).

### Loneliness, health and lifestyle outcomes by age group

Table 3 shows the multivariable adjusted associations between loneliness and physical and mental health or lifestyle outcomes, stratified by age group. We observed statistically significant effect modification by age group and loneliness for self-perceived health, visiting a physician within the past year, BMI and smoking, but not for any other health or lifestyle characteristic (all other p-interactions ≥ 0.05; data not shown). The association of loneliness with self-perceived health was strongest in the 30 to 59 year age group but was also statistically significant in 15 to 29 year old subjects as well as in subjects aged 60 years or older. Loneliness was not statistically significantly associated with BMI for the total sample, but younger lonely participants (up to 59 years) tended to have lower odds of being overweight or obese than older participants (Table 3). Lonely 15 to 29 and 30 to 59 year old subjects were statistically significantly more often ever or current smokers than subjects who never felt lonely. In the oldest age group, loneliness was most strongly associated with visits to a physician within the past year, followed by the middle-aged group.

**Table 3:** Multivariable adjusted associations between loneliness and lifestyle and health-related factors stratified by age group of the 2012 Swiss Health Survey^1^.

*Loneliness*	BMI^2^			Smoking^3^			Visit to a physician within the past year^4^			Self-perceived health^5^		
	Ref.	*OR*	*95% CI*	Ref.	*OR*	*95% CI*	Ref.	*OR*	*95% CI*	Ref.	*OR*	*95% CI*
Age groups (years)												
≥15 to <30	1	0.83	[0.67,1.03]	1	**1.21**	**[1.02,1.44]**	1	1.00	[0.82,1.23]	1	**1.98**	**[1.37,2.85]**
≥30 to <60	1	0.92	[0.82,1.03]	1	**1.11**	**[1.00,1.24]**	1	**1.32**	**[1.16,1.51]**	1	**2.19**	**[1.88,2.54]**
≥ 60	1	1.00	[0.86,1.16]	1	1.06	[0.91,1.24]	1	**1.80**	**[1.40,2.31]**	1	**1.64**	**[1.40,1.94]**
*P-Interaction*^6^			*0*.*026*	* *	* *	*0*.*004*	* *	* *	*0*.*004*	* *	* *	*0*.*044*

^1^ Weighted according to the Swiss general population and adjusted for sex, area of residence, nationality, educational level, marital status, household size and social support

^2^ Body mass index < 25.0 kg/m^2^ vs. ≥ 25.0 kg/m2

^3^ Never smokers vs. ever and current smokers

^4^ No vs. Yes

^5^ Good, very good vs. fair, poor, very poor

^6^ Interactions by age groups and loneliness for health outcomes assessed by Wald tests

Our sub-group analysis performed by including only the two extremes of loneliness (i.e. feeling never lonely vs. feeling very often lonely) revealed similar results (S1 Table).

## Discussion

One third of the Swiss population reported feeling lonely to some extent and about five percent claimed to feel lonely often or very often. Loneliness was more prevalent in young adults and in individuals older than 75 years. Although our results are merely descriptive, they are in accordance with a U-shaped association over the life course reported in earlier studies [3, 13], although we observed a peak of feeling lonely in the age ranges of 30 and 60 years. However, as observed by Heinrich and Gullone [33] many studies on the prevalence of loneliness were not up to date. The higher prevalence of loneliness in all age groups investigated in our study than in the mentioned survey on loneliness in 25 European nations [13] most probably reflect different categorizations of the term “loneliness” used in the two studies (feeling lonely all or almost all the time and most of the time in the European survey versus very often, quite often and sometimes in the SHS).

### Loneliness and physical and mental health

We observed in our analyses of the SHS data that participants who felt lonely were significantly more likely to report chronic diseases, hypercholesterolemia, diabetes, distress, and depression, poor self-rated health, and more visits to medical doctors than participants who did not feel lonely.

Participants who felt lonely to some extent in our study on the SHS data reported being affected by chronic diseases more often than individuals who did not feel lonely. Whether the report of chronic diseases is elevated in lonely people or whether chronic diseases are indeed more frequent in lonely people is not obvious in our study, due to the single item question about ongoing diseases or health problems lasting for at least 6 months or expected to last for longer than 6 months. Nevertheless, other studies also revealed a positive association of loneliness with chronic diseases [16, 58].

In contrast to some [21, 38] but not all other studies [59], we observed in the SHS no association between loneliness and hypertension. In a study of young adults [60], loneliness was associated with higher total peripheral resistance, a mechanism by which loneliness may contribute to the development of hypertension [59].

Loneliness was also associated with psychological distress in the present SHS data. Other studies found similar results, going into even more detail, finding for example that the association of loneliness with distress depended on the duration of experienced loneliness or that associations were only observed for specific dimensions of distress [61–63].

Loneliness was additionally associated with depression in our analyses of Swiss data. In the literature, depression has often been described to be accompanied by loneliness, but loneliness does not inevitably end in depression. Thus, loneliness may be both a risk factor for or a consequence of depression. Our findings are in line with those of other studies that found positive associations between loneliness and depression [43, 64, 65]. Although there are not only cross-sectional but also longitudinal studies that found an increased risk of depression with precedent loneliness, many of these studies included only older adults [43, 66, 67]. There are also a number of studies on children [68–70], adolescents [71–73] and younger adults [74, 75]. But less studies explored the association of loneliness with depression in middle-aged adults [47]. Thus, our results contribute to a better awareness of an underestimated health risk factor for all ages, and not only among older adults.

Similar to the present study on SHS data, several other studies found loneliness to be associated with poor self-rated health [16, 26, 28, 76]. In a longitudinal study in Finland, never or seldom feeling lonely predicted good self-rated health over time [37]. The association was stronger among women than among men. The authors of the study hypothesized that, among men, voluntary work and the associated social support might reflect pathways between loneliness and self-reported health. Among women, the initial significant association disappeared after adjusting for baseline health conditions. In our study, the association was not modified by sex but it was by age. Even though a significant association was observed in all three age groups, the association of loneliness with self-perceived health was surprisingly strongest in 30 to 59 year olds.

In our study, lonely individuals visited a medical doctor more often during the past year than participants who did not feel lonely. This association was observed in middle-aged and older but not younger individuals, which confirms results of previous studies [77–79]. Overall, lonely individuals might need to see a medical doctor more often because of their poor health. Furthermore, as Ellaway et al. [79] pointed out medical doctors possibly fulfill a social role for those who need someone to talk to.

Furthermore, our analyses of the SHS data, revealed no overall association between loneliness and BMI although the association was modified by age, pointing towards a tendency for lower odds of overweight and obesity in lonely younger but not in older participants. Our findings are in contrast to evidence from the literature of a positive association between loneliness and overweight, obesity and central obesity. Accordingly, in a cross-sectional study on 1289 adults in Australia a higher proportion of lonely than non-lonely individuals were overweight and obese [46]. In a population-based study in England, loneliness was associated with an increased likelihood for meeting criteria of the metabolic syndrome and with the individual criteria of central obesity and elevated fasting blood glucose, but not of high blood pressure and dyslipidemia [19]. As pointed out in the literature, higher food consumption might partially explain the association of loneliness with central obesity [19, 59, 80]. It was hypothesized by Jaremka et al. [81] that loneliness might predict higher post-brandial ghrelin concentration and hunger in women with a lower BMI. Ghrelin, a hormone which increases appetite, may thus link loneliness to weight gain. One explanation for our negative results in relation to loneliness and high body weight in the SHS may be the more frequent visits to medical doctors by lonely than by not lonely individuals. Indeed, medical doctors may have given advice to overweight patients to lose weight.

### Loneliness and lifestyle factors

In our analyses of the SHS data, feeling lonely was associated with smoking, physical inactivity, and non-adherence to the recommendation of fruit and vegetable consumption. Sex did not–with one exception–modify these associations. Age, on the other hand, had a modifying effect for smoking.

Our results in relation to smoking are partly in line with a newer systematic review, which found half significant and another half non-significant associations between loneliness and smoking [82]. In our study, lonely young (15 to 29 year old) and middle-aged (30 to 59 year old) participants, but not those of 60 years and older, smoked more often than participants who did not feel lonely. As DeWall and Pond [83] pointed out, young individuals may start smoking in an attempt to connect with others and gain social acceptance.

We observed no association between loneliness and neither chronic alcohol consumption nor binge drinking in our analyses of the SHS data. A population-based study in the US found an association of loneliness with reduced alcohol use, and not with binge drinking in individuals of 50 years or older [84]. In contrast, loneliness was considered to be a contributing, maintaining and poor prognostic factor in the development of alcohol abuse and a risk factor for all stages of alcoholism in a recent review [85]. Another study tried to explain these inconsistent findings by assessing transient loneliness and individual drinking behaviors, revealing that loneliness was related to an increase in solitary consumption and a decrease in social alcohol consumption [86]. Unfortunately, we were not able to confirm this potential explanation for the inconsistent association between loneliness and alcohol consumption because the SHS does not include data on social and solitary alcohol consumption.

For physical activity, we observed an association between loneliness and physical inactivity, independently of age. To our knowledge, no study had yet examined the association of loneliness with physical activity in the age range of total adulthood. But our findings are in accordance with previous literature on older adults, such as an Israeli study with a random sample [87] and a Canadian longitudinal study [88], in which loneliness was associated with a lower odds of engaging in physical activity. Similarly, in the study of Hawkley et al. [44] loneliness was an independent risk factor for physical inactivity among adults aged 50 years and above. As Hawkley & Cacioppo pointed out, loneliness is associated with poor emotional self-regulation [3], which may contribute to reduced motivation to engage in physical activity. Further studies, especially with samples of younger individuals, are needed to elucidate these associations.

We observed no overall association between feeling lonely and diet awareness in our analyses of the SHS data, but lonely men did mention diet awareness more often than male participants who did not feel lonely. Adherence to the recommendation of fruit and vegetable consumption, on the other hand, which can be interpreted as a proxy for “healthy” diet [56], was reported less frequently among lonely individuals in our study. To our knowledge the association of loneliness with healthy or unhealthy diet has scarcely been examined, with the exception of the effect of loneliness on malnutrition in the elderly [89] and in relation to eating disorders [90, 91]. However, our data were too limited to further evaluate these associations.

Overall, Hawkley and Cacioppo [3] proposed a model that explained how loneliness has health consequences and how it influences health behaviors. According to this model, loneliness is accompanied by feelings of hostility, stress, pessimism, anxiety, etc. and represents a dispositional tendency that activates or modifies genetic, neurobiological and immune functioning. In addition, loneliness is associated with a diminished capacity for self-regulation, which is of importance in relation to lifestyle behaviors. All of this contributes to adverse physical and mental health outcomes and adverse health behaviors.

### Strengths and limitations

Our study had two major strengths: First, we included all adults in our large population-based survey, stratifying them by age, as most of the existing studies focused on adolescents or older adults [7, 14, 16–22, 24] rather than on the entire adult age range. Second, the associations of loneliness with various physical and mental health aspects were examined. Research on these associations, especially with behavioral factors, is still rare and only scarce research exists for younger adults. Furthermore, we tested for age and sex interactions, which have rarely been evaluated before [8, 92]. Unfortunately, we could not analyze the adolescents separately because of their limited number, and thus, included them in the group of younger adults. A further strength is the use of a validated scale for depression and psychological distress as well as the multivariable adjustment for our large sample, which gives a good population based estimate of distress levels and depression status in Switzerland. Last but not least, our study is representative of the Swiss population, although individuals living in institutions (e.g. nursing homes, hospital, prisons) were not included and thus, we missed a group of individuals with worse health, which are more frequent in older age groups. A further strength were the additional analyses including only the two extremes of feeling lonely (i.e. never vs. very often), which confirmed our results.

Results of our study also entail several limitations. First, we were limited by the cross-sectional study design. No causal conclusions could be derived since the direction of the associations cannot be determined. Thus, it is difficult to determine whether loneliness followed exposure in time or exposure resulted from loneliness. However, our results support prospective studies and are in line with theoretical frameworks on the association of loneliness and health. Due to the lack of longitudinal data in Switzerland and the limited research on loneliness and its overall consequences, it is still worthwhile to present population-based cross-sectional data. Furthermore, we dichotomized loneliness due to the fact that only 2.7% and 1.5% reported to feel lonely quite often or very often in, respectively, in two categories. However, for the comprehensibility a dichotomization is advantageous and also used in studies frequently [7, 23]. A further limitation is that the study was based on self-reported data, assessed only once. Inaccurate self-reporting can be caused by recall bias, social desirability bias and errors in self-observation. There might also be bias with regard to a healthy participant effect in the Swiss Health Survey. Furthermore, as Luhmann & Hawkley [14] pointed out, in the case of age, loneliness may be more readily reported in older age when it is considered age-typical than in younger age. Indirect measures reduce this bias by avoiding the use of the term “loneliness” etc. Indirect measures are also invariably multi-item measures that offer greater reliability than single-item measures. In the present study, loneliness was assessed by one single-item question, which included the word “lonely”. Single-item questions were considered valid compared to multi-item questions for the same construct, in some, but not in all studies. [93–95]. The use of a direct single-item question which included the word “lonely” may have resulted in under-reporting due to the social stigma of loneliness. Another consequence of this phenomenological approach is that participants may have understood loneliness in different ways. Other variables in our study, such as physical activity and body weight, were based on self-reported information, which is prone to recall bias. Lastly, due to the low overall response rate of 54% in the SHS, the possibility of response bias cannot be ruled out. The use of weighting factors, nevertheless, allows for the extrapolation of the results in relation to age, sex, region and nationality from the sample to the total population living in Switzerland [96].

## Conclusion

Loneliness was apparent throughout the total age range of adulthood and was associated with poorer physical and mental health and unhealthy behavior. Furthermore, loneliness was modified by age, but not sex (with one exception), for some but not all physical and mental health aspects. Our findings illustrate the importance of considering loneliness for physical and mental health and lifestyle factors, not only in older and younger, but also in middle adults. Due to our cross-sectional study design no causal relationship could be inferred and, thus, evaluating loneliness in a longitudinal perspective is needed to contribute to a better understanding of the importance of loneliness as an independent risk factor for physical and mental health in Switzerland.

### Ethics approval and consent to participate

The data collection and data storage for the Swiss Health Survey (Schweizerische Gesundheitsbefragung) does not require formal approval by an ethics committee. This data collection is specifically permitted under Swiss law (SR 431.012.1 and SR 431.112.1). Individuals invited to participate received a brief description of the study and could decline to participate or withdraw at any time. Participants’ responses were treated confidentially and aggregated anonymous responses were utilized for the analyses presented herein.

## Supporting information

S1 Table Associations between loneliness dichotomized into extreme groups (feeling never vs. quite/very often lonely) and lifestyle or health-related factors of the 2012 Swiss Health Survey^1^. (DOCX)
