# Supplementary material for: Loneliness is adversely associated with physical and mental health and lifestyle factors: Results from a Swiss national survey
Source: PLoS One. 2017 Jul 17;12(7):e0181442. doi: 10.1371/journal.pone.0181442 (PMC5513556; doi:10.1371/journal.pone.0181442)
Supplement: S1 Table — (DOCX) [file pone.0181442.s001.docx]

|  |  | unadjusted | | age and sex adjusted | | multivariable adjusted | |
| --- | --- | --- | --- | --- | --- | --- | --- |
|  |  | *OR* | *95% CI* | *OR* | *95% CI* | *OR* | *95% CI* |
| **Physical and mental health** | | | | | | | |
| Chronic disease^2^ | No | 1 |  | 1 |  | 1 |  |
|  | Yes | **2.24** | **[1.87,2.68]** | **2.38** | **[1.97,2.87]** | **2.43** | **[2.00,2.95]** |
| Hypertension | No | 1 |  | 1 |  | 1 |  |
|  | Yes | **1.12** | **[0.92,1.35]** | **1.24** | **[1.01,1.53]** | 1.21 | [0.97,1.50] |
| High cholesterol | No | 1 |  | 1 |  | 1 |  |
|  | Yes | **1.49** | **[1.21,1.85]** | **1.73** | **[1.37,2.20]** | **1.69** | **[1.33,2.13]** |
| Diabetes | No | 1 |  | 1 |  | 1 |  |
|  | Yes | **1.65** | **[1.15,2.35]** | **1.97** | **[1.37,2.84]** | **1.81** | **[1.27,2.57]** |
| Psychological distress^3^ | Low | 1 |  | 1 |  | 1 |  |
|  | Moderate, high | **18.49** | **[15.13,22.59]** | **17.84** | **[14.63,21.77]** | **15.28** | **[12.37,18.87]** |
| Depression^4^ | No | 1 |  | 1 |  | 1 |  |
|  | Yes | **9.77** | **[6.91,13.82]** | **11.36** | **[8.30,15.53]** | **11.18** | **[8.14,15.36]** |
|  |  |  |  |  |  |  |  |
| Self-perceived health | Good, very good | 1 |  | 1 |  | 1 |  |
|  | Fair, poor, very poor | **5.70** | **[4.73,6.87]** | **6.83** | **[5.57,8.37]** | **5.55** | **[4.47,6.89]** |
| Visit to a physician within the past year | No | 1 |  | 1 |  | 1 |  |
|  | Yes | **1.78** | **[1.35,2.35]** | **1.60** | **[1.22,2.10]** | **1.71** | **[1.29,2.27]** |
|  |  |  |  |  |  |  |  |
| Body mass index | BMI < 25.0 kg/m^2^ | 1 |  | 1 |  | 1 |  |
|  | BMI ≥ 25.0 kg/m^2^ | 1.02 | [0.86,1.23] | **1.22** | **[1.02,1.47]** | 1.19 | [0.98,1.45] |
|  | | | | | | | |
| **Lifestyle factors** | | | | | | | |
| Smoking status | Never and ever smokers | 1 |  | 1 |  | 1 |  |
|  | Current smokers | 1.17 | [0.97,1.40] | **1.29** | **[1.07,1.55]** | **1.21** | **[1.00,1.46]** |
| Chronic alcohol consumption^5^ | No | 1 |  | 1 |  | 1 |  |
|  | Yes | 0.72 | [0.49,1.07] | 0.77 | [0.52,1.15] | 0.76 | [0.51,1.15] |
| Binge drinking | No |  |  | 1 |  |  |  |
|  | Yes | 0.72 | [0.59,0.88] | 0.85 | [0.68,1.07] | 0.98 | [0.78,1.22] |
| Physical activity | ≥ 150 minutes per week | 1 |  | 1 |  | 1 |  |
|  | < 150 minutes per week | **2.11** | **[1.76,2.54]** | **2.03** | **[1.69,2.44]** | **1.71** | **[1.41,2.07]** |
| Diet awareness | Yes | 1 |  | 1 |  | 1 |  |
|  | No | 0.99 | [0.81,1.19] | 1.12 | [0.92,1.38] | 1.00 | [0.81,1.24] |
| Adherence to the recommendation of fruit and vegetable consumption | Yes | 1 |  | 1 |  | 1 |  |
|  | No | **1.50** | **[1.13,2.00]** | **1.86** | **[1.39,2.50]** | **1.54** | **[1.13,2.08]** |

^1^ Weighted according to the Swiss general population and adjusted for age, sex, area of residence, nationality, educational level, marital status, household size and social support

^2^ Ongoing disease or health problem lasting for at least 6 months or expected to last for longer than 6 months

^3^ Measured by the 5-item mental health index

^4^ Measured by the PHQ-9 in the written questionnaire

^5^ Ethanol ≥ 20 g/day for women, ≥ 40 g/day for men
